# Survival, resistance, and fitness dynamics of *Escherichia coli* populations after prolonged exposure to copper

**DOI:** 10.1093/emph/eoaf015

**Published:** 2025-07-02

**Authors:** Sada Boyd-Vorsah, Arturo Torres Ortiz, Sophia Pulido, Brian Bui, Pamela J Yeh

**Affiliations:** Department of Ecology and Evolutionary Biology, University of California, Los Angeles, CA, USA; Department of Biochemistry, Emory University School of Medicine, Atlanta, GA, USA; Department of Ecology and Evolutionary Biology, University of California, Los Angeles, CA, USA; Department of International Health, Johns Hopkins Bloomberg School of Public Health, Baltimore, MD, USA; Department of Ecology and Evolutionary Biology, University of California, Los Angeles, CA, USA; Department of Ecology and Evolutionary Biology, University of California, Los Angeles, CA, USA; Department of Ecology and Evolutionary Biology, University of California, Los Angeles, CA, USA; Santa Fe Institute, Santa Fe, NM, USA

**Keywords:** microbial evolution, metal resistance, copper stress, whole-genome sequencing

## Abstract

**Background and objectives:**

Copper is an essential micronutrient and a widely used antimicrobial, yet its widespread application may accelerate microbial resistance. We investigated how long-term copper (II) sulfate (CuSO₄) exposure drives genetic and phenotypic changes in *Escherichia coli*, focusing on survival, resistance mechanisms, and antibiotic cross-resistance.

**Methodology:**

Fifty *E. coli* populations were evolved for 55 days under progressively increasing CuSO₄ concentrations. Whole-genome sequencing (WGS) identified genetic adaptations, while phenotypic changes were assessed using minimum inhibitory concentration (MIC) and fitness assays across CuSO₄ and antibiotic gradients.

**Results:**

CuSO₄ imposed strong selective pressure, with only 16% of populations surviving prolonged exposure. Survivors exhibited up to eight-fold increases in CuSO₄ resistance, though some reverted to ancestral resistance levels when selective pressure was removed. Fitness assays showed that CuSO₄-selected populations maintained significantly higher fitness in high CuSO₄ environments than controls and ancestors (*P* < .001). WGS revealed diverse mutations in stress-response and metal-tolerance genes (*cusA*, *acrB*, *corA*, *fur*, and *ybhA*) without a single resistance signature. Although antibiotic cross-resistance was not observed, some CuSO₄-selected populations displayed elevated MICs for levofloxacin, colistin, trimethoprim, fosfomycin, and meropenem. Similar trends in controls suggest that additional factors, such as adaptation to laboratory media, also contribute to resistance.

**Conclusions and implications:**

CuSO₄ exerts strong and variable selective pressure on *E. coli* populations, promoting diverse resistance pathways through distinct genetic and physiological mechanisms. While some CuSO₄-selected strains exhibited increased antibiotic resistance, trends in controls highlight the complexity of resistance evolution. These findings emphasize the need to monitor copper-driven antimicrobial resistance.

## INTRODUCTION

Copper (Cu) is both an essential micronutrient and a potent antimicrobial, widely applied in agriculture, aquaculture, and healthcare [1, 2]. Its persistence in soil and water exerts selective pressures on microbial populations, driving copper resistance and potentially promoting antibiotic co-resistance [3, 4]. In the Swedish Boreal Forest, copper exposure increased resistance to both copper and tetracyclines [4]. These findings emphasize the ecological and clinical importance of understanding microbial responses to copper.

Historically, copper has been used for its antimicrobial properties [5–7]. Modern healthcare settings employ copper surfaces to reduce microbial loads, effectively lowering healthcare-associated infections [8–10]. For example, copper surfaces reduced Methicillin-resistant *Staphylococcus aureus* (MRSA) and Vancomycin-Resistant *Enterococci* (VRE) burdens by 96.8% without changes to cleaning protocols [8]. However, hospital copper concentrations are typically reported as surface percentages (e.g. 55%–100%) rather than absolute concentrations [7]. This makes it difficult to identify copper concentrations that are both effective against microbes and those that may contribute to the emergence of resistance in clinical settings.

In agriculture and aquaculture, copper is used as a disinfectant, fungicide, and growth promoter [11–13]. Copper surfaces reduce foodborne pathogens like *Listeria* on poultry [11], and copper supplements are substituted for antibiotics in livestock feed. However, studies on farms show that copper supplements can select for *Enterococcus* strains resistant to multiple antibiotics, raising concerns about the role of copper in driving antimicrobial resistance (AMR) in the environment [14]. Additionally, copper is used as a fungicide for plant disease prevention [13] and in aquaculture to prevent infections and algal growth [12].

Environmentally, copper concentrations vary significantly. Copper concentrations in surface waters affected by industrial activities can range from 0.0005 to 1 mg/l in the USA [15], while contaminated soil may reach levels greater than 1000 mg/kg, ~1500 mg/l [16]. In agricultural settings, particularly in viticulture, copper-based fungicides contribute to soil concentrations exceeding 100 mg/kg, with some long-established vineyards reporting levels as high as 3000 mg/kg [17]. Additionally, wastewater can contain copper concentrations as high as 10 000 mg/l, highlighting extreme contamination scenarios [18]. Given copper’s extensive use in healthcare and environmental contexts, understanding microbial adaptation to copper is critical for predicting resistance evolution and optimizing its use in infection control strategies.

Copper’s dual roles as a micronutrient and antimicrobial stem from its ability to generate oxidative stress, disrupt membranes, and interfere with iron–sulfur cluster assembly [19–21]. Bacteria counter copper stress through efflux systems (CusCFBA, CopA), detoxifying enzymes (CueO, superoxide dismutases), and regulatory proteins (CusRS, CueR) [22–24].

This study investigates how long-term exposure to copper (II) sulfate (CuSO₄) shapes the evolution of *Escherichia coli* populations. We specifically asked: (i) how does CuSO₄ exposure affect survival, resistance, and fitness dynamics?; (ii) what genetic changes accompany CuSO₄ resistance?; and (iii) whether CuSO₄ resistance leads to antibiotic cross-resistance? To answer these questions, we performed a 55-day evolution experiment with 50 *E. coli* populations under increasing CuSO₄ concentrations, followed by whole-genome sequencing (WGS) and phenotypic assays to characterize resistance dynamics and their implications for AMR. CuSO₄ contamination, whether from agricultural runoff or industrial waste, may drive the emergence of resistant microbial populations, complicating efforts to predict and manage resistance.

## METHODOLOGY

### Bacterial strain

We used *E. coli* strain BW25113, a derivative of K-12 [25]. Antibiotic resistance markers were excised, and ancestral resistance to CuSO₄ and antibiotics was confirmed by minimum inhibitory concentration (MIC) assays.

### Evolution experiment

**Figure 1:**
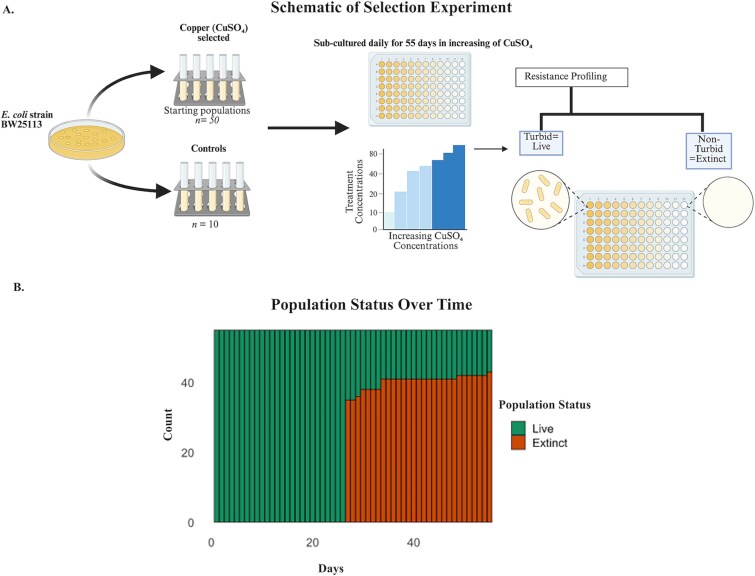
(A) Schematic of the selection experiment. (B) Survival of *E. coli* varies by population after 55 days of CuSO_4_ exposure as only 16% of CuSO_4_-selected populations remained viable, indicating a high extinction rate under CuSO₄ stress; which suggests that CuSO₄ imposes a strong selective pressure, potentially driving genetic or physiological adaptations necessary for survival in high-copper environments.

To investigate how CuSO₄ exposure influences the evolution of CuSO₄ resistance, we conducted a long-term experimental evolution study using *E. coli* BW25113 (Fig. 1A). *E. coli* was streaked onto Davis Minimal Broth (DMB) agar supplemented with 1% Thiamine HCl (hereafter referred to as DMB) and incubated overnight at 37°C with shaking at 220 rpm. Three independent single colonies were selected, grown in 5 ml DMB overnight, and used to determine the ancestral MIC. OD600nm measurements were taken using a Tecan Infinite M200 PRO Multimode Microplate Reader, and cultures were diluted to an OD600nm = 0.05. MICs were determined in 96-well plates containing 200 μl of diluted culture and DMB with two-fold serial dilutions of CuSO₄ ranging from 2500 to 0 mg/l. After 22–24 h incubation, ancestral MICs ranged from 78 to 312 mg/l.

Sixty independent colonies were randomly selected from fresh DMB agar plates. Ten populations were designated as controls, while 50 were exposed to CuSO₄ selection. Cultures were initiated at 10 mg/l CuSO₄ (~1/8 of the lowest MIC) and passaged daily. CuSO₄ concentrations were increased weekly up to 80 mg/l. Higher concentrations (90–100 mg/l) were tested but consistently resulted in slow growth, leading us to cap the experiment at 80 mg/l. Cultures were considered extinct if turbidity was absent after 24 h. The evolution experiment ran for 55 days, surviving populations were stored in 25% glycerol at −80°C.

### Minimum inhibitory concentration assays for CuSO₄ resistance

To assess the impact of long-term CuSO₄ selection on *E. coli* populations, we conducted CuSO₄ MIC assays at the end of the 55-day evolution experiment. Frozen glycerol stocks of all surviving CuSO₄-selected (*n* = 8) and control (*n* = 5) populations were revived on DMB agar and incubated overnight at 37°C. Approximately five colonies from each population were inoculated into 5 ml of DMB and incubated for 22–24 h. To maintain selection conditions, CuSO₄-selected populations were supplemented with 10 mg/l CuSO₄ during overnight growth. Additionally, three ancestral populations, derived from independent single colonies, were included in MIC assays to provide baseline resistance estimates. Resistance is demonstrated by higher MIC values than the ancestral MIC.

Cultures were diluted to OD600nm = 0.05 before inoculation into MIC assays. MIC assays were performed in 96-well plates containing 200 μl of diluted culture and DMB supplemented with a two-fold serial dilution of CuSO₄, ranging from 2500 to 0 mg/l. After 22–24 h at 37°C with shaking, OD600nm readings were taken using a Tecan Infinite M200 PRO Multimode Microplate Reader. MIC values were defined as the lowest CuSO₄ concentration at which OD600nm remained below 0.05 above the uninoculated media.

To assess resistance stability, CuSO₄-selected populations were passaged for 7 days in CuSO₄-free DMB (Day 62) before repeating MIC assays under the same conditions. Stability of resistance was defined as the maintenance of an elevated MIC relative to ancestral levels after the removal of selection pressure. All MIC assays were performed with three biological and two technical replicates per population.

### Growth and fitness assays

To assess growth dynamics under varying CuSO₄ concentrations, we conducted 24-h growth curve assays using a 384-well plate format. Three ancestral populations, five control populations, and eight CuSO₄-selected populations were revived from glycerol stocks and grown overnight in DMB at 37°C with shaking (200 rpm). CuSO₄-selected populations were maintained in DMB with 10 mg/l CuSO₄ to preserve selection pressure.

Overnight cultures were diluted to OD600nm of 0.05 and inoculated in duplicate into 384-well plates with a final volume of 80 μl per well. A two-fold CuSO₄ dilution series, matching MIC assay conditions, was used. OD600nm was measured hourly for 24 h with 20-s shaking intervals (Tecan Infinite M200 Pro).

Growth was defined as the net increase in OD600nm over time. To correct for CuSO₄-induced optical interference, OD600nm values were baseline-adjusted by subtracting the initial OD600nm (time = 0) for each well. Relative fitness was calculated by normalizing the OD600nm at each CuSO₄ concentration to its corresponding OD600nm at 0 mg/l CuSO₄ after 24 h: Relative Fitness = OD600nm at given CuSO₄ concentration/OD600nm at 0 mg/l CuSO₄. Values above 1 indicated a relative fitness advantage, while values below 1 indicated a cost of resistance.

### Antibiotic susceptibility assays

To evaluate the potential for collateral sensitivity and co-resistance in CuSO₄-selected *E. coli* populations, we conducted MIC assays across antibiotics with distinct mechanisms of action. The antibiotics tested included β-lactams (amoxicillin, meropenem), protein synthesis inhibitors (chloramphenicol, gentamicin), membrane disruptors (colistin), DNA synthesis inhibitors (levofloxacin, trimethoprim), and a cell wall synthesis inhibitor (fosfomycin).

MICs were determined for eight CuSO₄-selected and five control populations, which were revived from glycerol stocks and grown overnight in DMB at 37°C with shaking (200 rpm). To maintain selective pressure, CuSO₄-selected populations were supplemented with 10 mg/l CuSO₄. Overnight cultures were diluted to an OD600nm = 0.05 and inoculated into 96-well plates with two-fold serial dilutions of each antibiotic (final volume = 200 μl). Maximum concentrations were set at 8× the ancestral MIC [26]. MICs were defined as the lowest antibiotic concentration at which OD600nm remained below 0.05 above the uninoculated media background after 22–24 h of incubation.

Ancestral MICs, established prior to selection [26], were used as baselines and compared to EUCAST clinical breakpoints [27] to assess clinical relevance (Supplementary Table S1). MIC fold changes were calculated relative to the ancestral MIC for each antibiotic.

### DNA extraction and whole-genome sequencing

WGS was performed to identify genetic variants associated with CuSO₄ exposure, determine whether genetic changes occurred over time, and if changes were consistent across populations. To prepare samples for sequencing, each population was first cultured overnight in 5 ml of DMB media from 25% glycerol-preserved stocks. The overnight cultures were streaked onto DMB agar plates and incubated overnight to ensure that cells were viable, free from contamination, and exhibited consistent growth characteristics. A single colony from each plate was selected and cultured overnight in 5 ml of DMB media. The media used to culture the CuSO₄-selected strains was supplemented with a concentration of CuSO_4_ of at least 10 mg/l. Thus, the final sequencing was performed on individual clones from each evolved population. Afterward, the cultures were subjected to high-speed centrifugation, and the supernatant was discarded.

DNA extractions were performed using the Quick-DNA™ Fungal/Bacterial Miniprep Kit (Zymo Research, D6005), following the manufacturer’s protocol, including the addition of beta-mercaptoethanol to the genomic lysis buffer. DNA was sequenced using MinION (Oxford Nanopore) with an R9.4.1 flow cell. Native barcoding enabled multiplexing of samples, with 1 μg of genomic DNA per sample. Basecalling was performed using Guppy v6.4.8 with default parameters.

### Nanopore sequence analysis

Quality analysis of the long-reads was performed using NanoPlot and Filtlong (https://github.com/rrwick/Filtlong) [28]. Raw sequencing reads shorter than 1000 bp were removed from further analysis. Additionally, reads were filtered for read quality, removing the lowest 5% of reads. Filtered long-reads were assembled using Flye v2.9.3 [29] with default parameters. The resulting assembly was polished by mapping the long-reads back to the assembly with Medaka v2.0.1 (https://github.com/nanoporetech/medaka). Assembly quality was assessed using Quast [30] and Busco [31].

Assemblies were compared to the *E. coli* strain K-12 reference genome NC_000913. Each assembly was mapped against the reference genome using minimap2 with the asm5 option, after which genetic variants were inferred with paftools [32] and consensus sequences were created with the consensus function within BCFtools [33]. Coverage and base quality statistics were estimated by mapping the filtered long-reads back to the assembly using minimap2 v2.26 with the asm5 option. Positions covered by less than 30 reads and with a base and mapping quality lower than 10 were removed. Read frequency for a variant was defined as the number of reads supporting a variant divided by the total number of reads at that position. Any variant supported at a read frequency lower than 75% within a sample was considered ambiguous or too polymorphic to be associated with CuSO_4_ resistance, and thus it was discarded. The functional consequence of the identified variants was inferred using the csq algorithm of BCFtools [34]. Consensus sequences were concatenated and only variable sites among isolates were kept for further analysis. Small indels were detected using Clair3 [35] with default parameters. Large structural variants were detected using Sniffles2 [36], with default parameters and keeping only variants tagged as “PRECISE” by the software. Indels overlapping with homopolymer regions 2 bp or longer were removed. The final alignment was analyzed within R [37] using the package Ape [38]. A maximum likelihood phylogenetic tree was inferred using a GTR model as implemented within Phangorn [39]. Functional categories were assigned following the nomenclature used by COG (Clusters of Orthologous Groups) as annotated in the KEGG (Kyoto Encyclopedia of Genes and Genomes) and the Shigen PEC (Profiling of *E. coli* Chromosome) databases.

### Statistical analysis

All statistical analyses were performed in R (v4.2.3) [37] using Kruskal–Wallis tests, Wilcoxon rank-sum tests, ANOVA, and linear models. *P* < .05 was considered significant.

## DISCUSSION

### CuSO₄ imposes strong selective pressure and drives resistance

**Figure 2:**
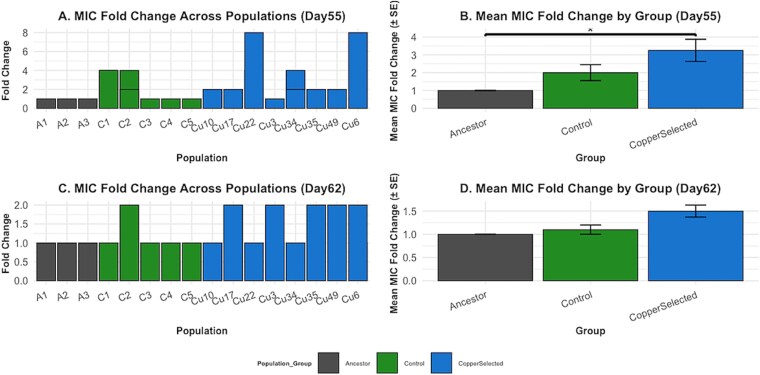
CuSO_₄_ resistance evolution and stability across *E. coli* populations. (A) MIC fold change across populations after 55 days of selection in CuSO₄-containing media; bars represent mean MIC values relative to the ancestral MIC, with colors indicating group identity: ancestor (“A”), control (“C”), and CuSO₄-selected (“Cu”).(B) A Kruskal–Wallis test revealed a significant difference between groups’ Mean MIC fold change (±SE) (*P < .05), and a Dunn’s post hoc test with Bonferroni correction indicated that MIC values were significantly higher in CuSO₄-selected populations compared to the ancestral populations after 55 days.(C) MIC fold change across populations after an additional 7 days of incubation in CuSO₄-free media to assess stability of resistance in the absence of selective pressure. (D) Mean MIC fold change (±SE) for each experimental group after 7 days in CuSO₄-free conditions.

After 55 days of selection, CuSO₄ imposed strong selective pressure, with only 8 of 50 CuSO₄-exposed populations surviving, while all control populations persisted (Fig. 1B). MIC assays revealed that CuSO₄-selected populations evolved resistance, exhibiting two- to eight-fold MIC increases relative to the ancestor (Fig. 2A). The highest resistance was observed in populations Cu6 and Cu22 (MIC = 2500 mg/l), while Cu3 retained ancestral-like resistance. However, C1 and C2 also demonstrated a four-fold increase in CuSO₄ MIC.

### Resistance stability following removal of CuSO₄ selection

After 7 days without CuSO₄ (Day 62), many CuSO₄-selected populations exhibited reduced MICs (Fig. 2C). Some reverted fully (Cu34, Cu22, Cu10), while others (Cu49, Cu35, Cu17) retained partial resistance. Cu3 unexpectedly increased its MIC after removal from the CuSO₄ environment. Most control populations maintained ancestral MICs levels, though some maintained moderate increases. Despite these reductions, the mean MIC fold change was not statistically significant after no CuSO₄ exposure (Fig. 2D), suggesting that resistance is reversible but varies across populations.

### CuSO₄-selected populations exhibit enhanced but variable fitness

**Figure 3:**
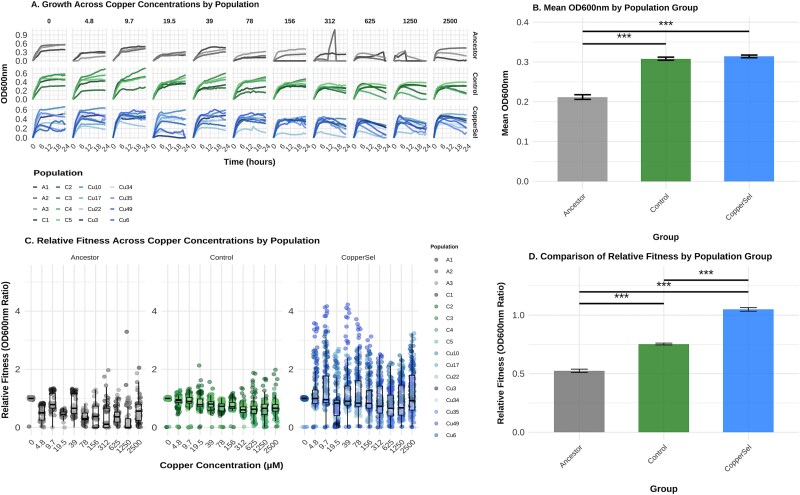
Bacterial growth and fitness of *E. coli* populations across CuSO₄ concentrations. (A) Growth curves of ancestor, control, and CuSO₄-selected populations over 24 h in increasing concentrations of CuSO₄ measured hourly by OD600nm in a 384-well plate. (B) Mean OD600nm values (± SE) for each population type at the end of the growth curve assays (***P < .001). (C) Relative fitness (ratio of fitness, calculated as OD600nm at a given CuSO₄ concentration normalized to OD600nm at 0 mg/l CuSO₄) of each population across CuSO₄ concentrations. (D) Mean OD600nm values (± SE) for fitness assays, which show both control and CuSO₄-selected populations exhibited significantly greater growth and relative fitness compared to Ancestors, with CuSO₄-selected populations showing the highest fitness at elevated CuSO₄ concentrations based on pairwise Wilcoxon tests.

Growth curve assays showed that CuSO₄ strongly influenced population fitness across copper gradients (Fig. 3). At low CuSO₄ concentrations (≤78 mg/l), all populations grew similarly. However, at intermediate concentrations (156–312 mg/l), CuSO₄-selected populations outperformed controls and ancestors, maintaining higher OD600nm (Fig. 3A and B). At high concentrations (>625 mg/l), growth was generally suppressed across populations, though some CuSO₄-selected populations displayed residual growth. Fitness trends (Fig. 3C and D) mirrored these patterns, with CuSO₄-selected populations consistently outperforming both ancestors and controls. Variation was evident: Cu49 showed consistently high fitness, while Cu3 and Cu6 performed poorly under high CuSO₄ stress. Statistical analyses confirmed significant group differences (*P* < 2e-16), with CuSO₄-selected populations showing significantly higher fitness than both controls and ancestors (*P* < .001). A linear model confirmed that CuSO₄ selection was necessary to sustain elevated fitness under copper stress (interaction term *P* = .0005).

### Antibiotic susceptibility shifts are variable and inconsistent

**Figure 4:**
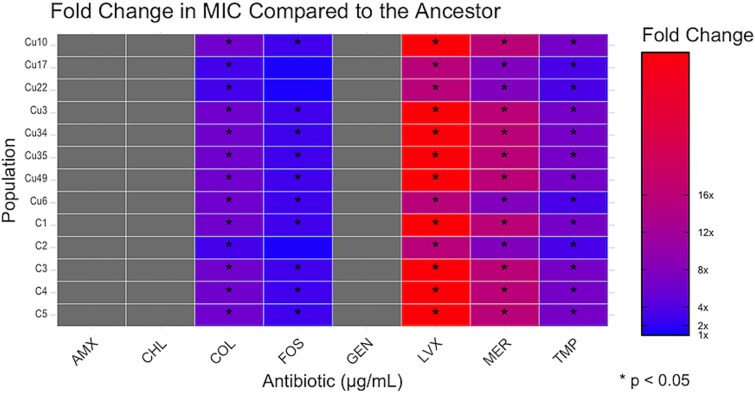
Heatmap displaying the fold change in MIC for eight antibiotics (amoxicillin [AMX], meropenem [MER]), protein synthesis inhibitors (chloramphenicol [CHL], gentamicin [GEN]), membrane disruptors (colistin [COL]), DNA synthesis inhibitors (levofloxacin [LVX], trimethoprim [TMP]), and a cell wall synthesis inhibitor (fosfomycin [FOS]) relative to the ancestral strain (*P < .05).

Antibiotic susceptibility profiles revealed no consistent pattern of cross-resistance or collateral sensitivity (Fig. 4). Both CuSO₄-selected and control populations showed variable MIC changes for colistin, trimethoprim, levofloxacin, fosfomycin, and meropenem, while susceptibility to amoxicillin, gentamicin, and chloramphenicol remained unchanged. MIC shifts were inconsistent among CuSO₄-selected populations, suggesting that these changes likely stem from general stress or media adaptation rather than copper exposure alone.

### Genetic basis of CuSO₄ resistance

**Figure 5:**
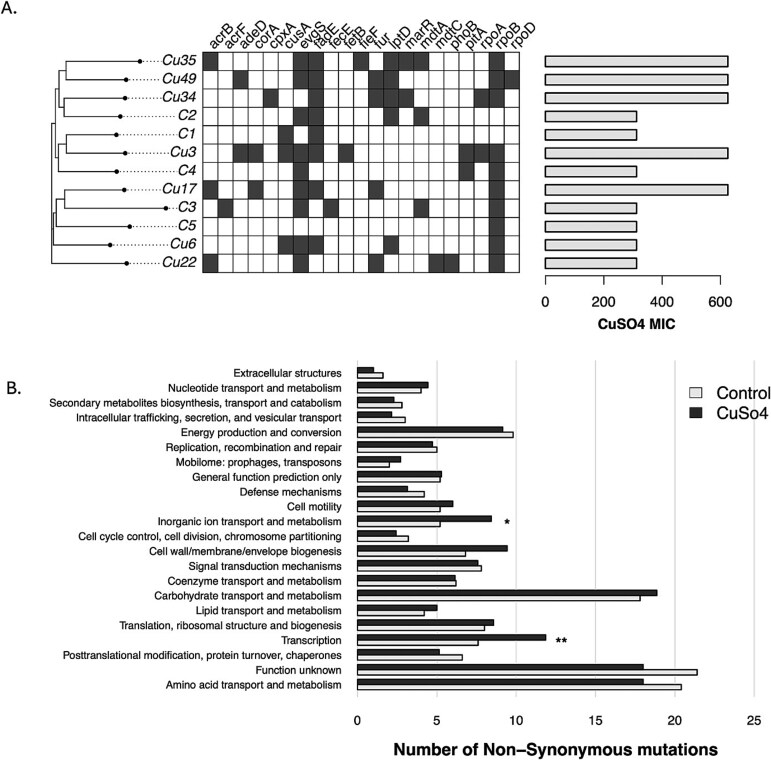
(A) Distribution of non-synonymous mutations across strains (Cu denotes samples grown in the presence of CuSO_4_ and controls grown in standard conditions are denoted by C). (B) Bar plot representing the average number of non-synonymous mutations in samples grown in CuSO_4_ and not grown in CuSO_4_ (*P < .001).

Genetic responses to CuSO₄ exposure, were performed using WGS of CuSO₄-exposed and control populations after 55 days. One population (Cu10) failed quality control and was excluded. After assembly, all genomes formed one or two contigs, with the largest contig nearly spanning the full length of the *E. coli* K-12 reference genome and showing a BUSCO completeness percentage near 90% for all samples (Supplementary Table S2). The alignment revealed 1270 polymorphic sites, including 783 non-synonymous mutations (Supplementary Table S3). Mutation counts were similar across populations, and there were no significant differences in total variants between the two groups (*P* > .7; Supplementary Table S4). Phylogenetic analysis showed no clear clustering of CuSO₄-exposed populations (Fig. 5A).

While most mutations occurred in both groups, 477 evolved exclusively in CuSO_4_-exposed isolates, including 297 non-synonymous and 228 singleton mutations. Notably, six CuSO₄-exposed populations acquired a non-synonymous mutation in *ybhA*, absent from all controls, while four populations grown in CuSO_4_ shared a *fur* missense mutation. Additional mutations were identified in genes linked to metal resistance, including *acrB*, *yehM*, *lptD*, *adeD*, and *corA*. Functional categorization showed enrichment of mutations in genes related to ion transport and metabolism (*P* = .008) and transcription (*P* = .028) (Fig. 5B).

We detected a total of 274 indels and structural variants (Supplementary Table S5). Due to the high error rate in small indel detection around homopolymers in nanopore data [40–42], especially for R9.1 flowcells [43], any indel falling within homopolymer regions of 2 bp or longer was removed. After filtering, 38 indels and structural variants remained (Supplementary Table S5), none of which fell within genes previously associated with metal resistance.

## CONCLUSION

### CuSO₄ exerts strong but variable selective pressure

Long-term CuSO₄ exposure imposed strong but variable pressure on *E. coli*, with only 16% of populations surviving after 55 days (Fig. 1). Survival likely required specific resistance mechanisms, while most lineages declined due to fitness constraints, consistent with reports of metal stress driving population loss [44]. Thus, these findings also highlight the dual role of CuSO₄ as both a selective agent for resistance and a significant driver of population extinction.

Surviving CuSO₄-selected populations exhibited two- to eight-fold MIC increases (Fig. 2A), with Cu6 and Cu22 achieving the highest resistance (MIC = 2500 mg/l), Cu3 retained ancestral-like resistance. Two control populations (C1 and C2) also showed MIC increases, likely due to spontaneous mutations or media adaptation [45, 46]. Most control populations retained ancestral MICs (~312 mg/l), reinforcing CuSO₄ as the primary selective driver, whereas CuSO₄-selected populations consistently exhibited elevated resistance. Statistical analysis supports this, with no significant MIC differences between controls and the ancestor (*P* = .303), suggesting that observed increases among controls fell within natural variation.

The strength and consistency of selection influenced evolutionary outcomes. Although populations were not exposed to CuSO₄ concentrations consistently above the ancestral MIC (80 mg/l), resistance still evolved in multiple lineages. This aligns with prior work showing that sublethal levels of antibiotics or metals can select for highly resistant genotypes through diverse adaptive pathways [24, 46–48]. Future work could explore whether higher or fluctuating CuSO₄ concentrations further shape resistance or extinction dynamics.

### Resistance is plastic and reversible

Resistance was plastic and often reversible. After 7 days without CuSO₄, many CuSO₄-selected populations reverted partially or fully to ancestral MICs. The variability in resistance stability suggests that populations relied on diverse resistance mechanisms, some of which incurred fitness costs under no-CuSO₄ conditions. Variability suggests that AMR may only be retained when it offers long-term fitness benefits, even under fluctuating conditions [49]. It also suggests that not all populations followed the same evolutionary trajectory. Further investigation into whether lineages that lost resistance carry mutations in regulatory versus structural resistance pathways could clarify the genetic basis of resistance stability.

### Enhanced fitness with variation across lineages

Fitness assays (Fig. 3C and D) showed that CuSO₄-selected populations outperformed controls and ancestors under elevated CuSO₄ concentrations (≥156 mg/l), while exhibiting similar growth at low CuSO₄ levels (≤78 mg/l). However, performance varied; some populations (Cu49) maintained high fitness across conditions, while others (Cu3 and Cu6) faltered under extreme CuSO₄ stress (>625 mg/l). Patterns indicate that CuSO₄ resistance arises through multiple evolutionary pathways, leading to diverse fitness outcomes shaped by selection pressure, genetic background, and trade-offs. This variation reflects a rugged adaptive landscape, where some populations achieve high-fitness peaks while others remain constrained by physiological or regulatory limits [50, 51].

### Genetic basis of CuSO₄ resistance

Genomic analysis provides further insight into the variability observed in fitness landscapes and the genes involved in CuSO₄ resistance. Interestingly, CuSO₄-selected populations lacked a defining set of mutations that separated them from controls, with no clustering in the phylogenetic tree based on CuSO_4_ MIC (Fig. 5A). Instead, non-synonymous mutations in CuSO₄-exposed populations were enriched in genes associated with transcription, ion transport, and metabolism (Fig. 5B). These genes include transcriptional regulators, metal-binding proteins, and translocating ATPases, all of which are expected to play roles in stress-response mechanisms. The presence of polymorphisms in multiple genes associated with CuSO₄ tolerance suggests that resistance arises through the accumulation of mutations across multiple pathways rather than a single conserved mechanism. Most of the identified genes—*phoB* [52], *acrB* [53], *fetB* [54], *cusA* [55], *marR* [56], *mdtA* [57], *mdtC*, *evgS* [58], *corA* [59], *cpxA* [24, 60], *fief* [61], *rpoD* [62], *rpoA* [48], and *rpoB* [63]—have been previously linked to metal tolerance or resistance. Several of these genes were highlighted in a laboratory evolution study on CuSO₄ resistance by Boyd et al. [24] and are specifically implicated in CuSO₄ resistance. Among these mutations, *cusA* stands out for its extensively documented role in CuSO₄ resistance mechanisms [55]. Some mutations were exclusive to isolates exposed to CuSO_4_, which could indicate CuSO₄ resistance pathways. Certain mutations (*cusA*, *acrB*, *corA*, *fur*, and *ybhA*) were enriched in CuSO₄-exposed isolates, but not universally present in all resistant populations, reinforcing the idea that CuSO₄ adaptation is driven by diverse evolutionary trajectories. These include genes previously associated with resistance to CuSO₄ and other metals, such as the genes *fur*, *acrB*, *lptD*, *adeD*, and *corA*, as well as genes not previously linked to metal tolerance, such as *ybhA*, or *yehM*. Most notably, the gene *fur* evolved in four isolates exposed to CuSO_4_. The Ferric Uptake Regulator (Fur) protein is linked to iron regulation and homeostasis, it has previously been associated with resistance to multiple metals and interacts synergistically with proteins associated with CuSO₄ resistance, such as CueR [64]. Similarly, the gene *ybhA* evolved in six of the isolates exposed to CuSO_4_, although it has not been linked to metal tolerance. Interestingly, since some mutations in these genes were not exclusive to CuSO₄-selected strains, it is plausible that they contribute to general stress-response pathways, while specific mutations are critical for conferring CuSO₄ resistance. Results highlight CuSO₄ resistance’s heterogeneous nature, with diverse genetic changes enabling varied adaptive strategies. Further genomic and transcriptomic studies are critical for determining whether these mutations confer direct resistance or provide broader stress-response advantages in fluctuating environments.

### Ecological and clinical relevance

Widespread use of copper in agriculture, aquaculture, healthcare, and wastewater treatment raises concerns about its role in shaping microbial resistance. Although CuSO₄ may not universally drive antibiotic cross-resistance, enrichment of efflux pump and stress-response genes suggest possible indirect links to antibiotic resistance. Given the mobility of resistance genes via horizontal gene transfer and mobile genetic elements, copper-rich environments could still serve as reservoirs for multidrug resistance [65]. Overall, we demonstrate that while CuSO₄ imposes strong selective pressure, it promotes heterogeneous evolutionary outcomes. Resistance is often reversible, context-dependent, and shaped by a rugged fitness landscape [66]. Fiindings highlight the need to monitor environmental and clinical settings for interactions between metal exposure and resistance evolution.

## Supplementary Material

Supplementary_Table_1_eoaf015

supp_tab2_(1)_eoaf015

supp_tab3_Final_eoaf015

supp_tab4_Final_eoaf015

supp_tab5_eoaf015

## Data Availability

All raw sequencing data generated as part of this study have been submitted to the European Nucleotide Archive (ENA) and the NCBI Sequence Read Archive (SRA) under accession PRJEB87397.
