# Supplementary material for: Survival, resistance, and fitness dynamics of Escherichia coli populations after prolonged exposure to copper
Source: Evol Med Public Health. 2025 Jul 2;13(1):176–87. doi: 10.1093/emph/eoaf015 (PMC12409786; doi:10.1093/emph/eoaf015)
Supplement: Supplementary_Table_1_eoaf015 [file supplementary_table_1_eoaf015.docx]

Supplementary Table 1. Summary of Ancestral MICs and EUCAST Resistance Breakpoints. Ancestral MICs were performed previously in our lab (Mira et al., 2022). EUCAST Resistance Breakpoints were taken from European Committee on Antimicrobial Susceptibility Testing (EUCAST, Version 15.0, 2025). Ancestral MIC values were compared to EUCAST clinical breakpoints for *E. coli* to assess whether the tested concentrations are considered clinically significant.

| Antibiotic | Abbreviation | Ancestral MIC (μg/ml) | EUCAST Resistance Breakpoint (R > X mg/L) | Interpretation |
| --- | --- | --- | --- | --- |
| Amoxicillin | AMX | 4.8 | R > 8 | Susceptible |
| Chloramphenicol | CHL | 419.9 | MIC > 16* | Resistant |
| Colistin | COL | 0.32 | R > 2 | Susceptible |
| Fosfomycin | FOS | 0.70 | Interpretation not provided** | Likely Susceptible |
| Gentamicin | GEN | 26.4 | R > 2 | Resistant |
| Levofloxacin | LVX | 0.064 | R > 1 | Susceptible |
| Meropenem | MER | 0.125 | R > 8 | Susceptible |
| Trimethoprim | TMP | 0.29 | R > 4 | Susceptible |

Note:

1. For Chloramphenicol, EUCAST provides a screening cut-off of MIC >16 mg/L for resistance. Given the ancestral MIC of 419.9 μg/ml, this indicates the strain was already resistant at baseline.

2. For Fosfomycin, EUCAST does not provide explicit MIC breakpoints, but based on previous clinical literature, the ancestral MIC of 0.70 μg/ml suggests susceptibility.
